# Electrical resistivity imaging data for hydrogeological and geological hazard investigations in Taiwan

**DOI:** 10.1016/j.dib.2023.109377

**Published:** 2023-07-07

**Authors:** Ping-Yu Chang, Yonatan Garkebo Doyoro, Ding-Jiun Lin, Jordi Mahardika Puntu, Haiyina Hasbia Amania, Lingerew Nebere Kassie

**Affiliations:** a Department of Earth Science, National Central University, Taoyuan, Taiwan; b Department of Applied Geology, School of Natural Sciences, Adama Science and Technology University, Adama, Ethiopia; c Earth System Science, Taiwan International Graduate Program (TIGP), Academia Sinica, Taipei, Taiwan; d Department of Geology, School of Earth Sciences, Bahir Dar University, Bahir Dar, Ethiopia

**Keywords:** Electrical resistivity imaging, Hydrogeological characterization, Hydraulic parameter, Land subsidence, Geological structure

## Abstract

This data article presents electrical resistivity imaging (ERI) data and inverted models with the objectives of hydrogeological characterization, land subsidence studies, and geological structural detections in Taiwan. The ERI data for hydrogeological studies includes 5 ERI profiles from Changhua, 33 from Yunlin, 36 from Yilan, 23 from Taichung, 23 from Chiayi and Tainan, and 23 from Taipei basins. In addition, time-lapse ERI profiles are presented for 10 ERI from Yilan, 10 ERI from Pingtung, 11 ERI from Taichung, and 31 ERI from Minzu basins. Moreover, 10 ERI data were used to detect the Rusui Fault, 12 for the Qishan Fault, 13 for the Yuli Fault, and 25 for the Shanyi Fault. This data article contains 265 ERI profiles with a total survey length of 59,905 m. A single ERI profile contains hundreds to thousands of subsurface apparent resistivity data points. The data was collected between 2010 and 2022 from different regions of Taiwan. The main findings from the ERI data consisted here were reported by Lin et al. [1] for the Pingtung basin, Chang et al. [2] for the Minzu basin, and Jordi et al. [3] for the Taichung basin in order to estimate hydraulic parameters and characterize the aquifer systems. The ERI data presented here can be used for a variety of hydrogeological, geological, engineering, and environmental applications, and it can be further interpreted using machine learning and statistical methods. Therefore, the ERI data will helps in various subsurface applications, academic research, and educational purposes.

**Specifications Table**

**Table:** 

Subject	Geophysics
Specific subject area	Electrical resistivity imaging
Type of data	Figure Chart KML KMZ
How the data were acquired	The resistivity imaging data were collected by deploying multi-electrode resistivity instruments in specific survey profiles, injecting electric current into the ground, and measuring the variation in resistivity response in the subsurface material.
Data format	Raw Inverted Model
Description of data collection	The resistivity imaging data for the Yilan, Minzu, Taichung, and Pingtung sites were collected in different months to account for the dry and wet seasons, whereas data from other sites were collected in a single survey. The data repository contains detailed data descriptions for each survey site.
Data source location	Site/region: Taipei, Yilan, Minzu, Taichung, Yunlin, Chiayi, Tainan, Pingtung, and Taitung. Country: Taiwan
Data accessibility	Repository name: Mendeley data Data identification number: 10.17632/nkskkcpdg9.2 Direct URL to data: https://data.mendeley.com/datasets/nkskkcpdg9/2
Related research article	D.-J. Lin, P.-Y. Chang, J. M. Puntu, Y. G. Doyoro, H. H. Amania, L.-C. Chang, Estimating the Specific Yield and Groundwater Level of an Unconfined Aquifer Using Time-Lapse Electrical Resistivity Imaging in the Pingtung Plain, Taiwan, Water. 15(2023):1184. https://doi.org/10.3390/w15061184

## Value of the Data

• The ERI data from the various survey sites can be used to select sites for hydrogeological investigations, identify geological rock units, detect hazardous geological structures, and characterize engineering sites.
• The ERI data can be used to monitor groundwater levels by comparing them with future survey findings.
• The ERI data can be jointly inverted and interpreted with different geophysical data to obtain more reliable subsurface information. Other studies effectively integrated ERI data with seismic [4], electromagnetic [5] and ground penetrating radar [6].
• Using open-source inversion algorithms, raw ERI data can be reprocessed to generate 2D and 3D inverted models. Several studies [7], [8], [9], [10], [11] have successfully used open-source algorithms for ERI data inversion. Machine learning and statistical algorithms can be used to further interpret the inverted resistivity data. ERI data for several studies [12], [13], [14] have also been made available.

## 1 Objective

The primary focus of this work data was hydrogeological, land subsidence, and geological structural investigations such as aquifer characterization, groundwater level detection, groundwater recharge zone identification, hydraulic parameter estimation, clay layer identification, and fault detection [[1], [2], [3], [15]]. The hydraulic parameter was estimated, and groundwater levels were monitored using time-lapse ERI measurement data from the Yilan, Minzu, Taichung, and Pingtung alluvial fans. To determine groundwater potential recharge zones, single survey ERI data from alluvial fans in Taipei, Yunlin, Yilan, Chiayi, and Tainan were collected. The ERI data were collected to detect geological structures such as Titung's Rusui and Yuli Faults, Tainan's Qishan Fault, and Taichung's Shanyi Fault.

## 2 Data Description

The electrical resistivity imaging data for hydrogeological and geological hazard investigations conducted in various Taiwan locations are presented. The data can be found at Mendeley's data repository [15]: https://data.mendeley.com/datasets/nkskkcpdg9/2. The repository is divided into two major folders: ERI Data for Geohazard Detection and ERI Data for Hydeogeological Investigation. The ERI Data for Geohazard Detection is organized into four folders: 1 Ruisui Fault, 2 Qishan Fault, 3 Yuli Fault, and 4 Shanyi Fault. ERI Data Folders for Hydeogeological Investigation, on the other hand, include 1 Changhua ERI, 2 Yunlin ERI, 3 Yilan ERI, 4 Pingtung ERI, 5 Taichung ERI, 6 Minzu ERI, 7 Chaiyi and Tainan ERI, and 8 Taipei ERI. Tables 1 and 2 contain detailed data descriptions. Each survey site folder includes a detailed data description; for example, Table 2 shows ERI data descriptions for the Yunlin Chousui River Middle Alluvial Fan. Table 1 shows the total number of survey locations, the number of ERI profiles, and the survey's scope. The data collected by the 4-point light 10W LGM Lippmann resistivity meter is presented in 'URF' format, whereas the data collected by the AGI SuperSting R1 is presented in 'STG' format. If necessary, the ResIPy open-source software [8,10] can be used to convert the file to another format. Inverted resistivity models for each ERI profile are also presented in 'JPG' format. Google Earth KML and KMZ files containing the location of ERI profiles are also provided for each survey site.

**Table 1:** Describes the survey site, data collection year, profile length and total survey length, survey type, and purpose.

Survey site	Survey year	Number of ERI	Profile length (m)	Total profile length (m)	Survey round	Purpose of the survey
Changhua	2023	5	≈ 310	1550	Single-survey	Land subsidence
Yunlin	2022	10	370	3700	Single-survey	Artificial recharge site selection
	2021	23	100	2300	Single-survey	Land subsidence
Yilan	2020	10	100	1000	Time-lapse (Four-times)	Aquifer characterization
	2013	36	≈ 250	8980	Single-survey	Delineation of groundwater potential recharge zones
Pingtung	2019	10	≈ 150	1500	Time-lapse (Five-times)	Aquifer characterization
Taichung	2018	11	≈ 95	1045	Time-lapse (Five-times)	Aquifer characterization
	2015	23	≈ 360	8370	Single-survey	Delineation of groundwater potential recharge zones
Minzu	2019	9	≈ 115	1040	Time-lapse (Five-times)	Aquifer characterization
	2018	9	100	900	Time-lapse (Five-times)	Aquifer characterization
	2017	13	≈ 100	1300	Time-lapse (Five-times)	Aquifer characterization
Chiayi and Tainan	2016	23	≈ 290	6690	Single-survey	Delineation of groundwater potential recharge zones
Taipei	2014	23	≈ 365	8400	Single-survey	Delineation of groundwater potential recharge zones
Rusui Fault	2015	10	≈ 286	2860	Single-survey	Geological fault detection
Qishan Fault	2015	12	≈ 247	2965	Single-survey	Geological fault detection
Yuli Fault	2015	13	≈ 297	3855	Single-survey	Geological fault detection
Shanyi Fault	2013	25	≈ 138	3450	Single-survey	Geological fault detection

**Table 2:** Shows an example of ERI data descriptions for Yunlin: year of data collection, profile center coordinate, elevation, array types, electrode spacing, profile length, file types and names, and data collection instrument type.

Profile line name	Survey date (dd/mm/year)	Profile center coordinates		Elevation (m)	Profile orientation	Array type	Electrode spacing (m)	Profile length (m)	File type	File name	Type of instrument
		Longitude	Latitude								
ERI01	26/04/2022	23.733068°	120.420171°	24	S→N	WN	10	370	STG	ERI01 WN	SuperSting
ERI01	26/04/2022	23.733068°	120.420171°	25	S→N	SN	10	370	STG	ERI01 SN	SuperSting
ERI02	25/04/2022	23.721849°	120.390926°	18	S→N	WN	10	370	STG	ERI02 WN	SuperSting
ERI02	25/04/2022	23.721849°	120.390926°	19	S→N	SN	10	370	STG	ERI02 SN	SuperSting
ERI03	25/04/2022	23.719941°	120.376520°	17	W→E	WN	10	370	STG	ERI03 WN	SuperSting
ERI04	26/04/2022	23.727735°	120.347322°	13	S→N	WR	10	370	STG	ERI04 WR	SuperSting
ERI04	26/04/2023	23.727735°	120.347322°	14	S→N	SN	10	370	STG	ERI04 SN	SuperSting
ERI05	27/06/2025	23.681923°	120.349302°	13	S→N	WN	10	370	STG	ERI05 WN	SuperSting
ERI05	27/06/2025	23.681923°	120.349302°	14	S→N	SN	10	370	STG	ERI05 SN	SuperSting
ERI06	31/05/2022	23.6928075°	120.352592°	13	S→N	WN	10	370	STG	ERI06 WN	SuperSting
ERI07	27/04/2022	23.675901°	120.406282°	20	S→N	WN	10	370	STG	ERI07 WN	SuperSting
ERI07	27/04/2022	23.675901°	120.406282°	21	S→N	SN	10	370	STG	ERI07 SN	SuperSting
ERI08	27/04/2022	23.657532°	120.367433°	14	S→N	WN	10	370	STG	ERI08 WN	SuperSting
ERI08	27/04/2022	23.657532°	120.367433°	15	S→N	SN	10	370	STG	ER08 SN	SuperSting
ERI09	31/05/2022	23.653398°	120.343024°	12	S→N	WN	10	370	STG	ERI09 WN	SuperSting
ERI10	01/06/2022	23.623346°	120.377084°	14	W→E	WN	10	370	STG	ERI10 WN	SuperSting

WN denotes the Wenner-Normal array, WR denotes the Wenner-Reverse array, and SN denotes Schlumberger-Normal (SN).

Furthermore, the ERI data are described in terms of data collection time (dd/mm/year), profile start, center, and ending coordinates, survey site elevations, profile orientation, array type, electrode spacing, profile length, file types, and names, and the type of resistivity meter used for each survey area. For example, Table 2 shows ERI data descriptions for the Yunlin Chousui River Middle Alluvial Fan.

## 3 Experimental Design, Materials and Methods

### 3.1 Survey sites and data distribution

**Fig. 1:**
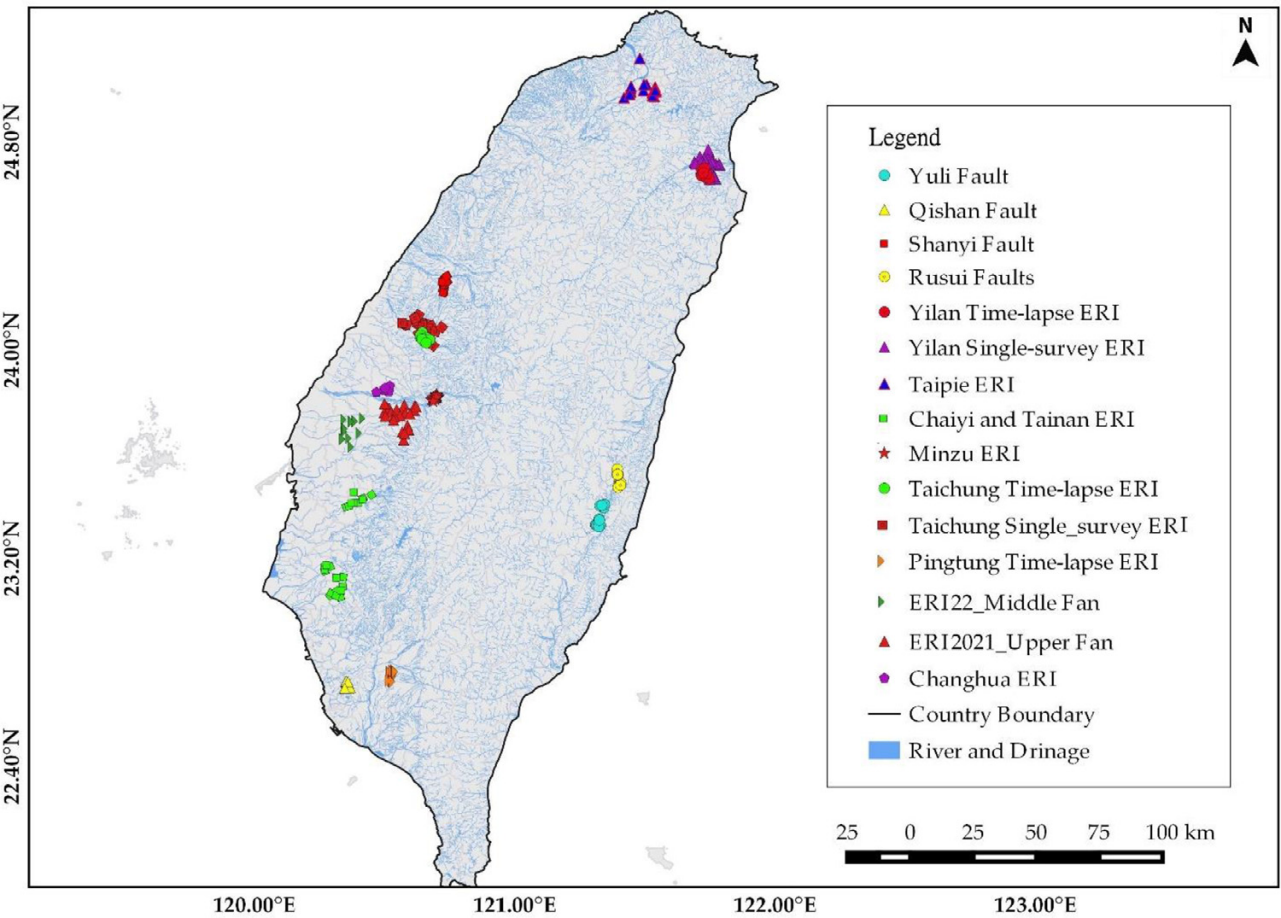
ERI data distribution for hydrogeological and land subsidence studies.

**Fig. 2:**
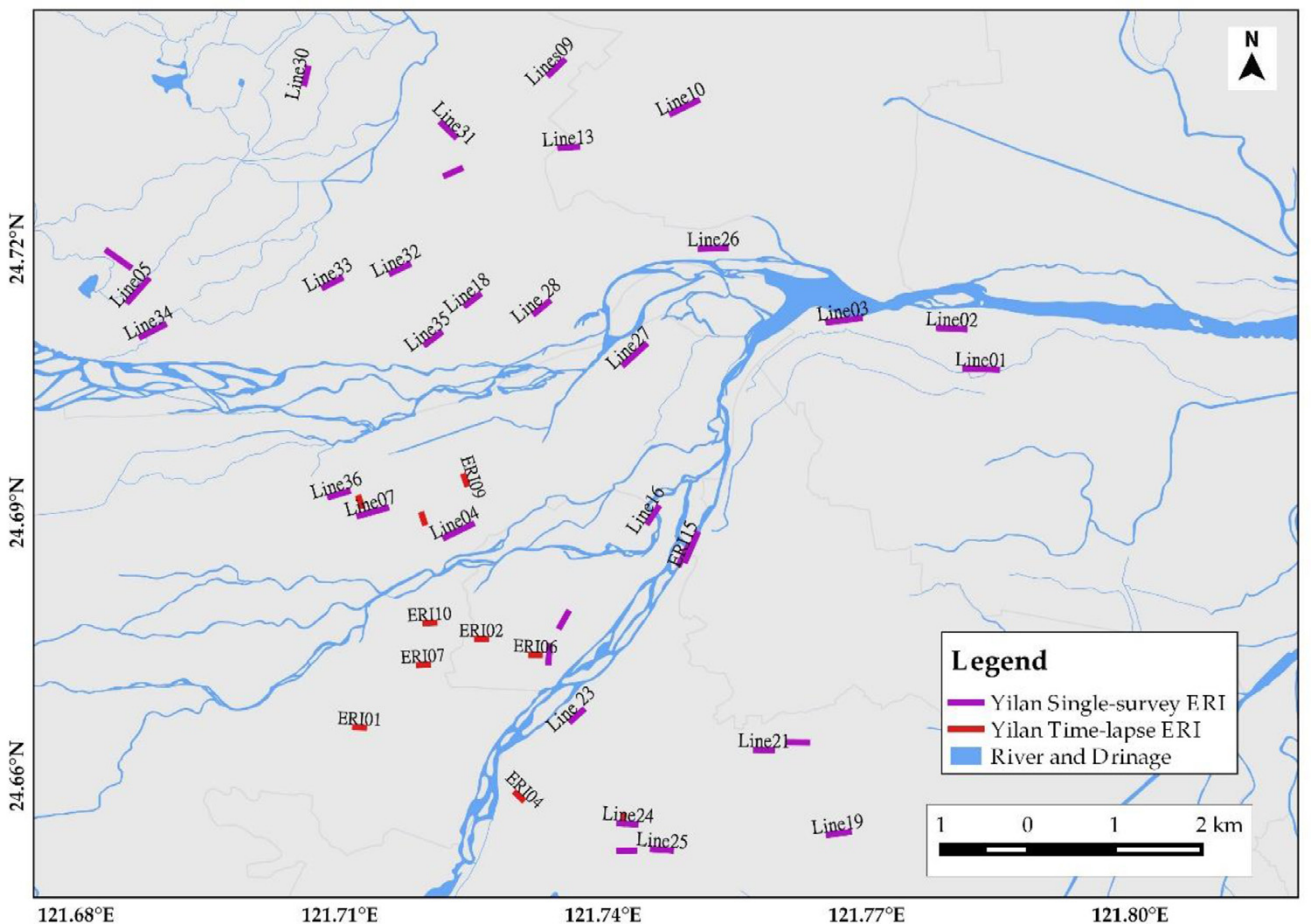
ERI data distribution of Yilan Alluvial Basin hydrogeological characterization.

**Fig. 3:**
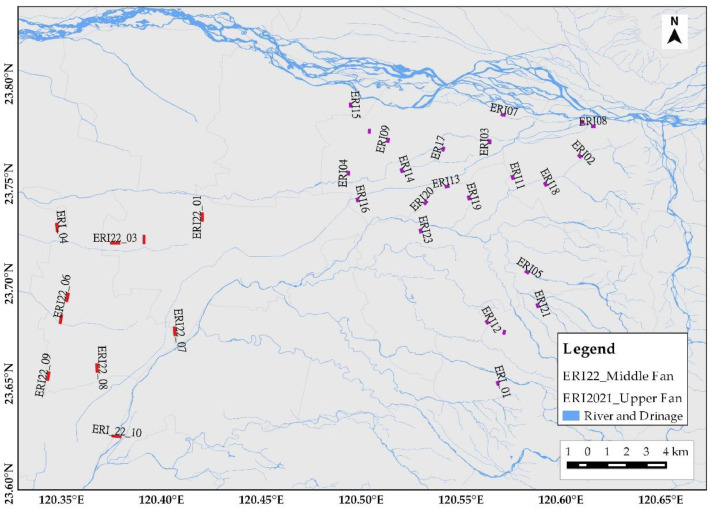
ERI data distribution for clay layer detection and artificial recharge site selection in Yunlin Alluvial Basin.

**Fig. 4:**
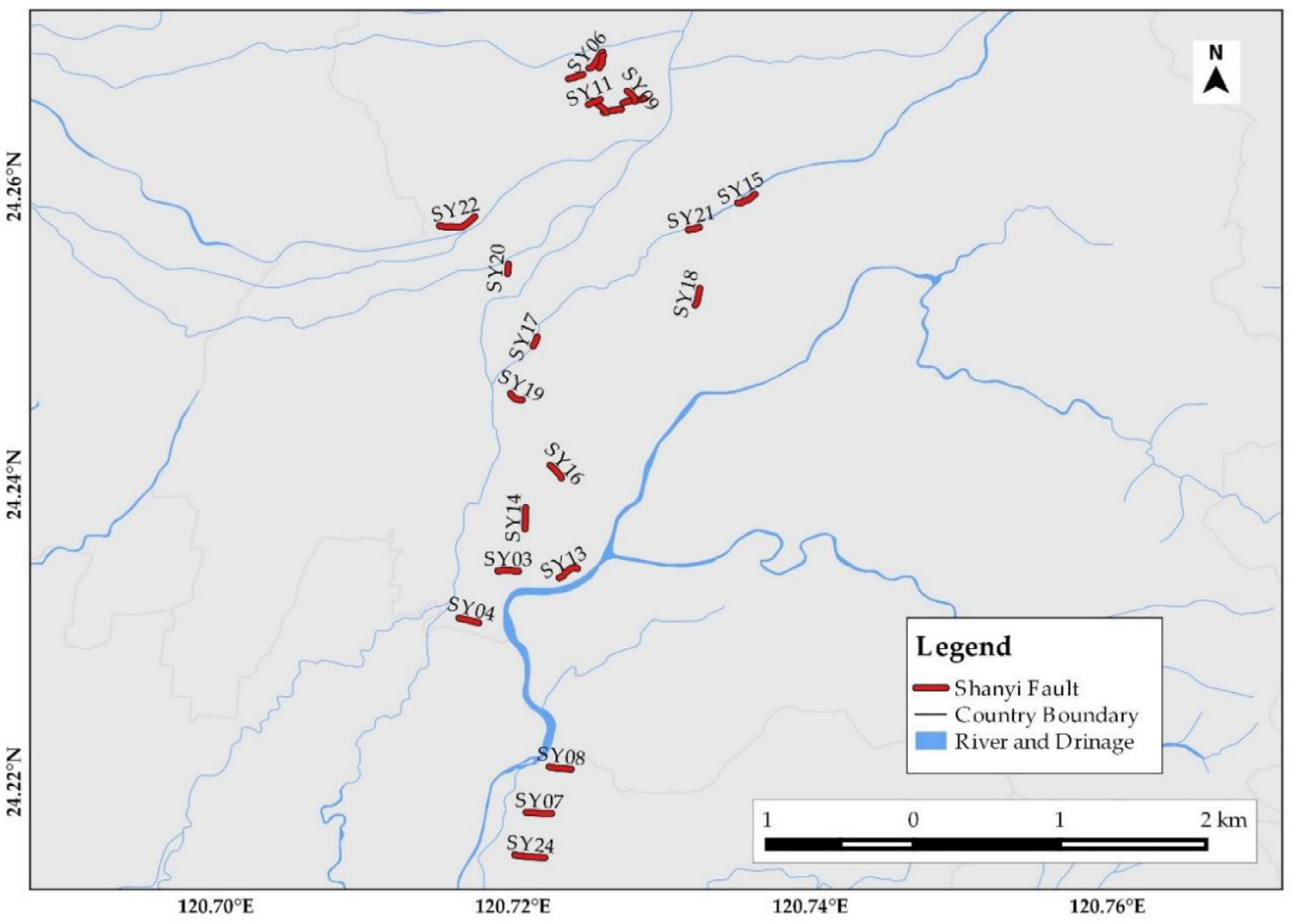
ERI data distribution for Shanyi fault detection around the Taichung area.

Fig. 1 depicts the overall ERI profile distributions for hydrogeological, land subsidence, and geological structural studies. It has 5 ERI from Changhua, 33 from Yunlin, 36 from Yilan, 23 from Taichung, 23 from Chiayi and Tainan, 23 from Taipei, 10 for Rusui Fault, 12 for Qishan Fault, 13 for Yuli Fault, and 25 for Shanyi Fault from a single survey. The time-lapse ERI profiles were collected from 10 ERI in Yilan (four time-lapse), 10 ERI in Pingtung (five time-lapse), 11 ERI in Taichung (five time-lapse), and 22 ERI in Minzu (five time-lapse), considering seasonal rainfall variation from Taiwan Central Weather Bureau Meteorological Observatory precipitation record. Yilan ERI data were collected for four months in 2020: February with 40 mm of monthly precipitation, May with 124.2 mm of precipitation, July with 56.0 mm of precipitation, and October with 621.0 mm of precipitation. Pingtung ERI data were collected over five months in 2019: February, May, July, September, and November, with precipitation of 1.0 mm, 5.2 mm, 19.6 mm, 7.1 mm, and 2.3 mm, respectively. Taichung ERI data were collected for five months in 2018: February (22.5 mm), May (73.0 mm), July (347.0 mm), September (20.0 mm), and October (7.5 mm). Furthermore, Minzu ERI data for five different months were collected: January, March, May, June, and September, with monthly precipitation of 10.1 mm, 8.3 mm, 5.0 mm, 16.1 mm, and 6.3 mm, respectively. Three survey sites, for example, are enlarged to show site-specific ERI profile distribution. Fig. 2 shows ERI data distribution for hydrogeological characterization of the Yilan Alluvial Basin. The Yunlin ERI data distribution for artificial groundwater recharge site selection and clay layer detection for land subsidence are presented in Fig. 3. Besides, Fig. 4 displays the geological structural or fault detection in the Shanyi area, Taichung.

### 3.2 Data collection

**Fig. 5:**
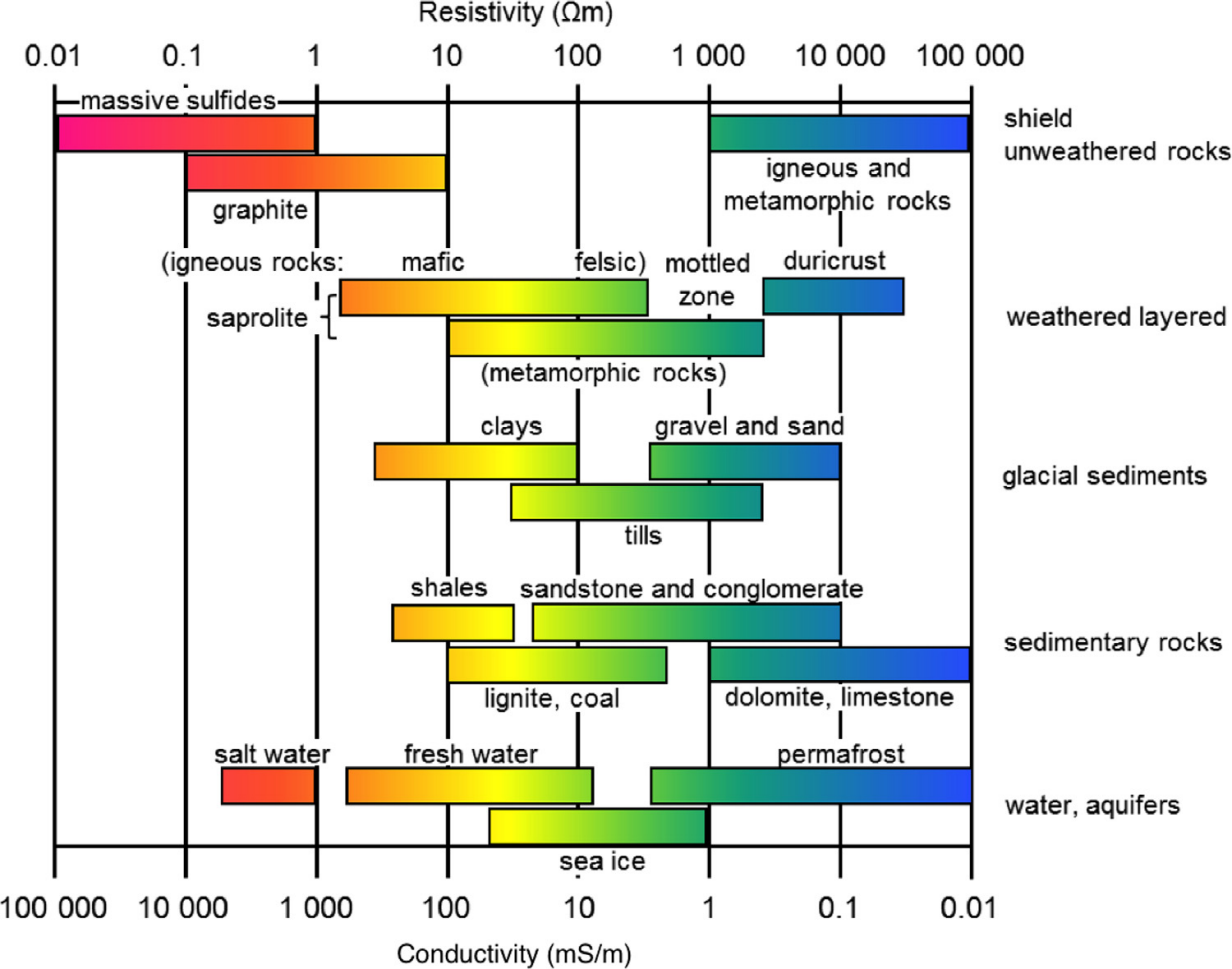
Electrical resistivity/conductivity proporties of common geological materials [16].

The 2D resistivity imaging data were collected by galvanically injecting a low-frequency electrical current into the ground via two current electrodes and measuring the voltage difference between two potential electrodes. Variations in resistivity values caused by the flow of electric current through various subsurface mediums can be used to identify unknown materials. Electrical resistivity of the subsurface material is related to the nature of soil composition (particle size distribution, mineralogy), structure (porosity, pore size distribution, connectivity), fluid content, concentration of dissolved electrolytes, clay contents, and temperature [16]. Fig. 5 depicts the electrical resistivity/conductivity characteristics of common subsurface geological materials.

**Fig. 6:**
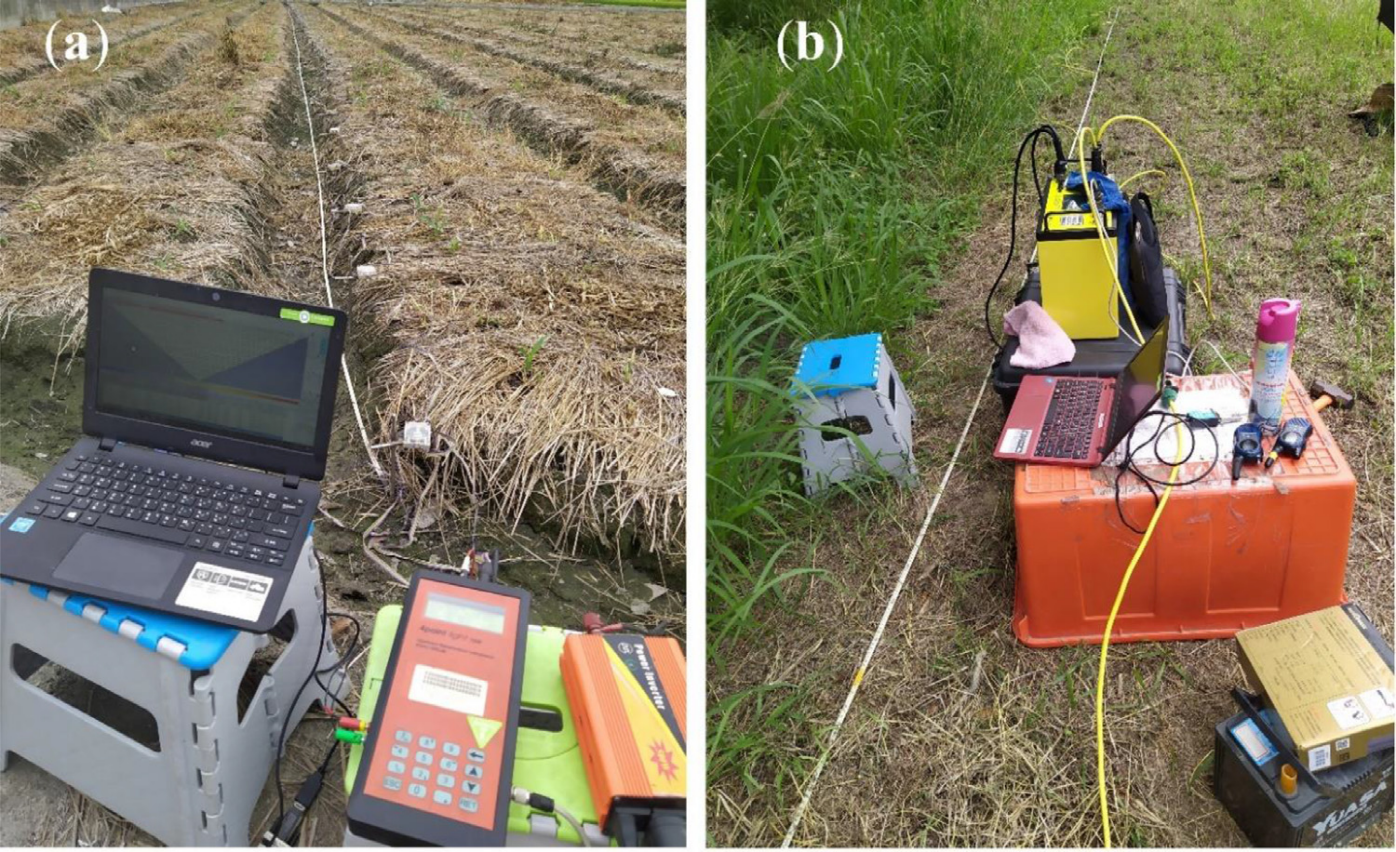
Acquiring ERI field data with multi-electrode (**a**) 4-point light 10W LGM Lippmann, and (**b**) AGI SuperSting R1 resistivity meters.

**Fig. 7:**
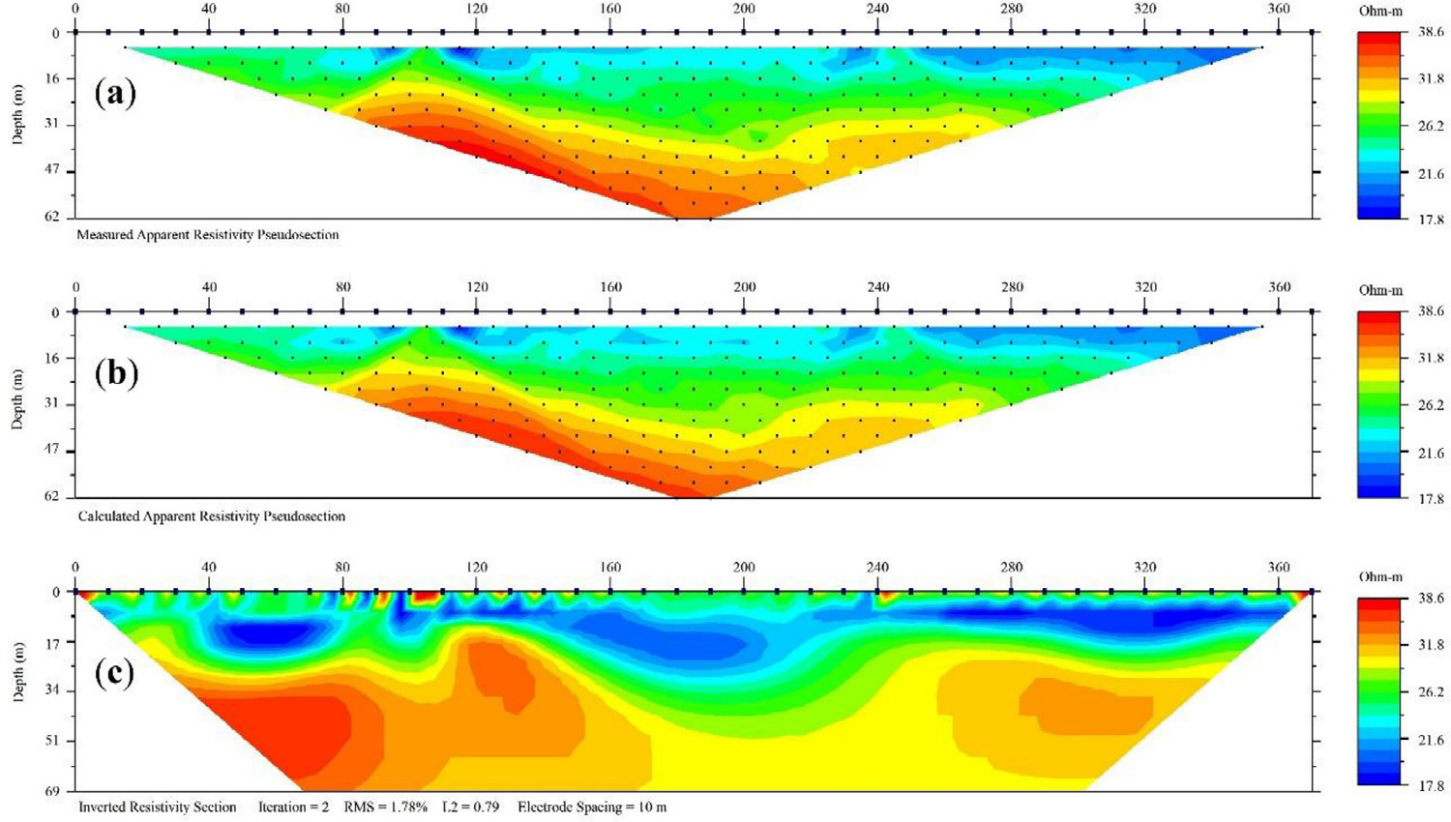
ERI data distribution and inversion models: (**a**) apparent resistivity data, (**b**) calculated apparent resistivity data, and (**c**) an inverted resistivity model.

The advanced multi-electrode resistivity meters were used to measure hundreds to thousands of data points in a single ERI profile by automatically changing the current and potential electrodes. Wenner, Schlumberger, and dipole-dipole arrays were used to collect resistivity data in the normal (forward) and reverse survey directions. Fig. 6 depicts the acquisition of field ERI data with a 4-point light 10W LGM Lippmann (Fig. 6a) and an AGI SuperSting R1 (Fig. 6b) resistivity meter. Electrodes were placed along the profiles and connected to cables with connector boxes, which were then connected to a resistivity meter during resistivity measurements. The electrodes were then tested for contact resistance before data collection, and apparent resistivity was measured. Then, as shown in Fig. 7a, hundreds to thousands of apparent resistivity data points can be measured for a single ERI profile.

### 3.3 Data processing and inversion

**Fig. 8:**
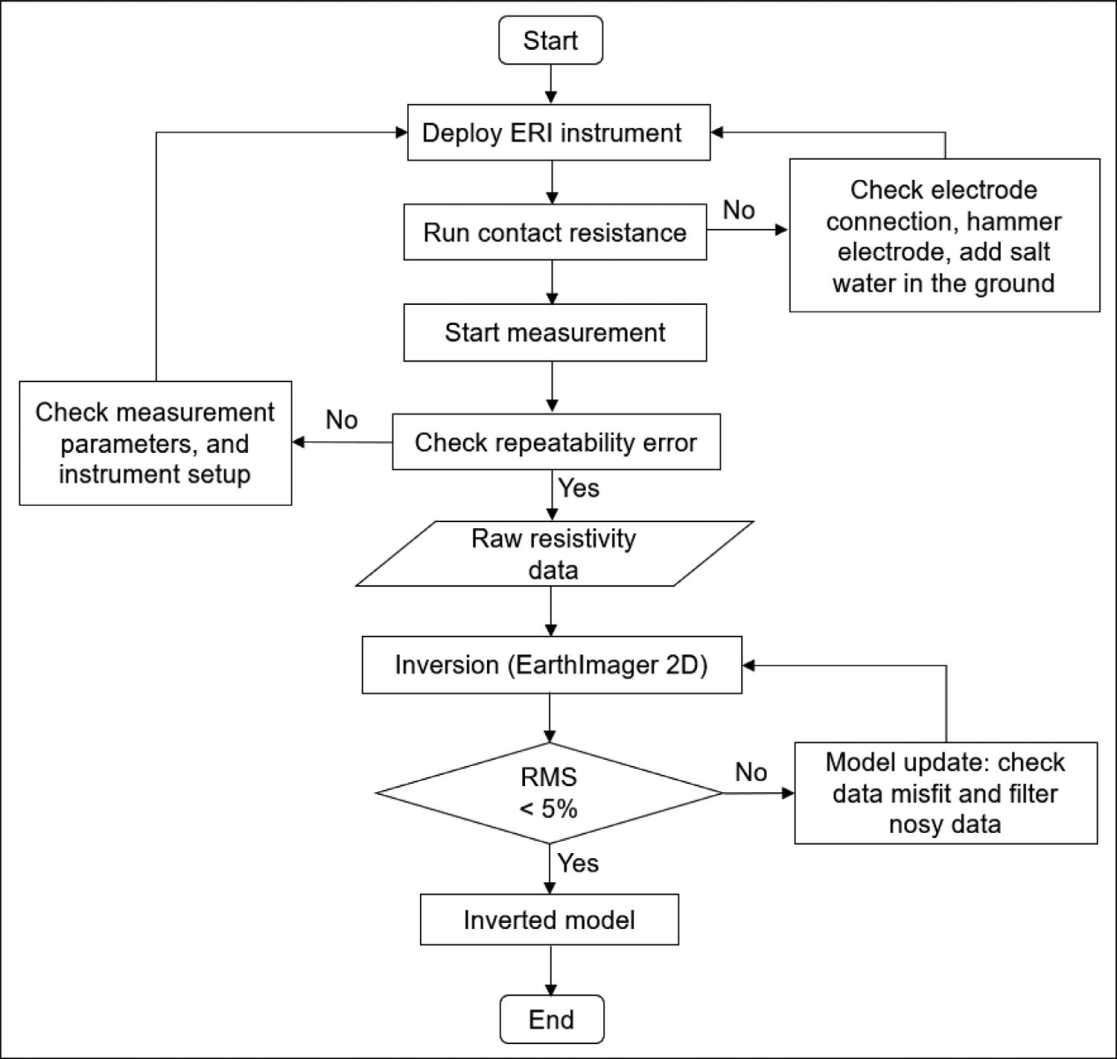
Flow chart of resistivity imaging data acquisition, processing, and inversion.

The measured data was filtered to remove noise. The EarthImager 2D software package [17] was used for data processing and inversion. Depending on the expected subsurface features, the commonly used smoothness-constrained least-square [18] and robust inversion [19] algorithms were used. The ERI data were mostly inverted using a smoothness-constrained least-square inversion algorithm. Iterative inversion was used until the misfit between measured and predicted resistivity data reached acceptable levels, where the root mean square (RMS) error is less than 5%, which may exceed for surveys in hard rock and noisy environments. For example, we show the ERI data distribution and inversion models (Fig. 7) for resistivity data collected from the Yunlin Chousui River Alluvial Fan. The data was collected using a Wenner array with electrode spacing of 10 m. Fig. 7a shows the distributions of apparent resistivity data, Fig. 7b shows model calculated apparent resistivity data, and Fig. 7c shows an inverted resistivity model. The overall ERI data acquisition, processing, and inversion approaches are depicted in Fig. 8.

## Ethics Statements

The authors declare that they have no known ethical issues in respect of the data reported in this article.

## CRediT Author Statement

**Ping-Yu Chang:** Conceptualization, Modification, Validation, and Writing- Original Draft. **Yonatan Garkebo Doyoro:** Conceptualization, Modification, Data Collection, Writing- Original Draft. **Ding-Jiun Lin, Jordi Mahardika Puntu**, and **Haiyina Hasbia Amania, Lingerew Nebere Kassiea**: Data Collection and Processing**.**

## Declaration of Competing Interests

The authors declare that they have no known competing financial interests or personal relationships that could have appeared to influence the work reported in this paper.

## Data Availability

Electrical resistivity imaging data for hydrogeological and geological hazard investigations in Taiwan (Original data) (Mendeley Data).
